# Estimating Prevention-Effective Adherence to HIV Pre-exposure Prophylaxis (PrEP) Among Australian Gay, Bisexual and Queer Men and Non-binary People: A Mixed-Methods Analysis

**DOI:** 10.1007/s10461-025-04899-1

**Published:** 2025-11-11

**Authors:** James MacGibbon, Anthony K. J. Smith, Timothy R. Broady, Sarah K. Calabrese, Jeanne Ellard, Dean Murphy, Tina Gordon, Simin Yu, James Gray, John de Wit, Martin Holt, Benjamin R. Bavinton

**Affiliations:** 1 Centre for Social Research in Health, UNSW Sydney, Sydney, NSW Australia; 2 School of Population Health, Curtin University, Perth, WA Australia; 3 Collaboration for Evidence, Research and Impact in Public Health, Curtin University, Perth, WA Australia; 4 Department of Psychological and Brain Sciences, George Washington University, Washington, DC USA; 5 Australian Research Centre in Sex, Health & Society, La Trobe University, Melbourne, Australia; 6 School of Population Health, UNSW Sydney, Sydney, NSW Australia; 7 New South Wales Ministry of Health, Sydney, NSW Australia; 8 Health Equity Matters, Sydney, NSW Australia; 9 Kirby Institute, UNSW Sydney, Sydney, NSW Australia; 10 Department of Interdisciplinary Social Science, Utrecht University, Utrecht, Netherlands

**Keywords:** Men who have sex with men (MSM), Gay and bisexual men (GBM), HIV prevention, Attitudes, Australia, Prevention-effective adherence, PrEP adherence

## Abstract

Oral HIV pre-exposure prophylaxis (PrEP) is highly effective when taken appropriately at times of HIV risk, termed “prevention-effective adherence”. To understand suboptimal adherence, we refined a brief measure for surveys among gay, bisexual and queer men and non-binary (GBQ+) people. We used a mixed-methods design, comprising a national, online cross-sectional survey (June–July 2023) and cognitive interviews (August–October 2023). Logistic regression identified characteristics of PrEP users who reported condomless anal intercourse with casual partners (CLAIC) that was not protected by their own PrEP use because they missed PrEP doses (“PrEP-unprotected CLAIC”). Cognitive interviews investigated whether the prevention-effective adherence measure was comprehensible. Of 2,046 survey respondents, 792 current PrEP users who had CLAIC in the past 6 months were included (Median age = 37, 86.7% gay, 34.5% non-daily-PrEP users). Of PrEP users who reported any CLAIC, 194 (24.5%) reported any PrEP-unprotected CLAIC. They were more likely to: be < 30 years old, be born in Asia vs. Australia, be part-time vs. full-time employed, use non-daily PrEP, have recently initiated PrEP, have experienced side effects from PrEP, and report recent sexualized drug use. They were less likely to find it easy to get PrEP. The 14 interviewees asked about the survey items (Mean age = 33; 50% gay; ) were able to answer the questions and reliably interpret the content. This first national estimate of prevention-effective adherence found that 24.5% of PrEP users who had CLAIC reported any PrEP-unprotected CLAIC. Targeted interventions in subgroups with more frequent PrEP-unprotected episodes must address side effects and other adherence barriers.

## Resumen

La profilaxis preexposición oral (PrEP) es altamente eficaz cuando se toma durante períodos de riesgo de VIH, lo que se denomina adherencia efectiva para la prevención. Refinamos una medida breve de adherencia para encuestas en hombres gais, bisexuales, queer y personas no binarias (GBQ+), y evaluamos sus características asociadas con relaciones sexuales anales sin condón con parejas ocasionales (RSAO) no protegidas por PrEP. En una encuesta nacional en línea (junio-julio 2023), 792 personas usuarias actuales de PrEP que reportaron RSAO fueron analizadas. El 24.5% reportó al menos una RSAO no protegida por PrEP. Estas se asociaron con menor edad, nacimiento en Asia, empleo parcial, uso de PrEP no diaria, inicio reciente, efectos secundarios, consumo sexualizado de drogas y dificultades para acceder a PrEP. Entrevistas cognitivas (*n* = 14) confirmaron comprensión del ítem. Las intervenciones deben centrarse en barreras de adherencia y considerar formulaciones de PrEP de acción prolongada adaptadas a preferencias individuales.

## Introduction

HIV pre-exposure prophylaxis (PrEP)—the regular use of antiretroviral medications—is highly effective at reducing the risk of acquiring HIV [1–3]. To ensure its effectiveness, PrEP must be taken appropriately during times of potential risk, a concept referred to as ‘prevention-effective adherence’ [4]. Unlike HIV treatment, PrEP does not necessitate continuous dosing to maintain its efficacy. Since 2019, on-demand PrEP use, also known as ‘2-1-1’ or event-driven PrEP, has gained popularity, increasing to nearly one third of PrEP-using gay and bisexual men in Australia [5, 6]. PrEP users may also choose to use PrEP daily for discrete periods of time (e.g., during travel or Pride festivals), known as ‘periodic PrEP’ [7]. Accurate, resource-efficient assessments of prevention-effective adherence must now account for diversity in PrEP dosing strategies among users and dynamic risk practices; that is, they must account for the timing of pill-taking alongside the timing of sexual behaviors presenting a risk of HIV acquisition. Prior assessments of PrEP adherence have not routinely accounted for recent sexual behavior and such assessments have been particularly challenging outside of tightly controlled trials and demonstration projects, which to date, have been the primary source of PrEP adherence data [8]. In this article, we assess the utility of a brief self-report measure that intends to overcome some of the challenges to measuring PrEP adherence outside of clinical trial settings. In doing so, we explore characteristics associated with suboptimal PrEP coverage among gay, bisexual and queer men and non-binary people in Australia.

Various methods have been employed to assess PrEP adherence, including self-report questionnaires [7, 9], pharmacological approaches using biological samples [10, 11], technological solutions such as electronic pill bottles [12], and surveillance methods like pharmacy dispensing or clinical records [4, 13]. While some of these methods can offer higher accuracy compared to self-reporting, they are often costly or complex to implement.

Self-report questionnaires are a simple option to measure prevention-effective adherence to PrEP, and can allow for the simultaneous collection of both pill-taking and sexual behavior data, but their accuracy can vary depending on the questionnaire design and the participant’s ability to recall details. These questionnaires typically inquire about various aspects of PrEP usage, such as current or recent use, dosing regimen (daily or on-demand PrEP), and any temporary breaks or discontinuation. However, solely tracking pill-taking is not sufficient to determine prevention-effective adherence, as it must also consider the individual’s HIV risk behaviors, particularly episodes of condomless anal intercourse. In Australia, condomless anal intercourse with casual partners (CLAIC) is regarded as the primary transmission route for HIV among gay and bisexual men [14], and this risk practice is therefore the focus of this paper. More broadly, assessing HIV risk in self-report questionnaires involves inquiring about factors like the number of sexual partners, their characteristics, the type of sexual activities, and condom usage during specified time periods (e.g., previous 6 months). Again, the problem here is that when asking about all sexual behaviors in a given time period, it is not possible to determine the timing of sex in relation to the timing of PrEP pill-taking.

In the past, methods for measuring prevention-effective adherence through questionnaires involved extensive questioning, often exceeding 20 questions [7, 15]. While effective, this approach is burdensome for both participants and researchers, potentially leading to reduced response quality and completion rates. In light of these challenges, there is a growing need for more efficient methods to measure prevention-effective adherence in both research and clinical settings. We devised a single item about PrEP coverage, derived from consultation with clinicians [16]. The item asked participants to report how many times in the last 6 months they had sex with a casual partner without a condom that was not ‘covered’ by the participant’s own use of PrEP, which was defined as missing more than one PrEP dose for daily PrEP use or any doses for ‘on-demand’ (2-1-1) PrEP users. This item was initially tested in a survey which included 409 PrEP users in Australia [16]. In that study, 46.5% of participants reported PrEP-unprotected CLAIC [16]. Participants were more likely to report any CLAIC not covered by their own PrEP use if they were younger, on a lower income, or employed less than full time [16]. These findings corroborate research on PrEP discontinuation, which suggests that younger people and those with lower incomes are more likely to take breaks or stop using PrEP [17]. Our previous research also showed that participants using non-daily PrEP and those who had experienced side effects were more likely to report CLAIC not covered by PrEP [16].

A weakness of the single-item approach to measuring prevention-effective adherence, as described by Fraser and colleagues [16], was that it could not account for the PrEP use or viral load status of the participant’s sexual partners. As others have argued, partners’ use of preventative strategies is important to consider as potential measures of ‘collective antiretroviral protection’ [18] or ‘prevention by proxy’ [19]. Additionally, although some explanatory text was provided to explain what was meant by the item phrasing, ‘not covered by PrEP’, it was unclear to what degree participants correctly understood the meaning or were able to (accurately) categorize their episodes of sex in the last 6 months as ‘covered’ or ‘not covered’. Fraser and colleagues (2024) recommended, in turn, that future research assess participants’ interpretation of the term ‘covered’. Further, it was suggested that a second item be paired with the question to examine partners’ HIV status and use of HIV prevention strategies; that is, to determine the degree to which those engaging in PrEP-unprotected CLAIC may be relying on their partners’ HIV status (e.g., serosorting), PrEP use or undetectable viral load to reduce HIV transmission risk.

Existing studies show that most methods of measuring prevention-effective adherence are challenging to implement in research studies and are impractical outside of clinical trials and demonstration projects. We therefore sought to adapt and examine our self-report ‘PrEP coverage’ item in a national Australian, cross-sectional survey data with a large sample of gay, bisexual and queer men and non-binary (GBQ+) people. In doing so, we identified characteristics associated with PrEP-unprotected CLAIC. Further, we checked GBQ + people’s understanding of the item and their ability to classify their own sexual practices using qualitative cognitive interviewing. Together, the current study evaluated the practicality of a brief measure of prevention-effective adherence that can be used to identify the specific education and health promotion needs of people who report suboptimal PrEP adherence. We also aimed to quantify the level of risk behavior for HIV acquisition among PrEP users, and to examine preferences for PrEP modalities among those with suboptimal PrEP adherence.

## Method

### Study Design and Participants

We used a concurrent, independent mixed-methods design to examine prevention-effective adherence to HIV PrEP among GBQ + people in Australia. A quantitative component, involving a cross-sectional survey, permitted broad analyses of characteristics associated with adherence to HIV PrEP and potential risk practices. A qualitative component, consisting of cognitive interviews, sought to contextualize the target population’s understanding of the survey item, and examine their ability to recall and classify their own sexual practices. By integrating the survey and interview findings, we take a ‘complementary strengths stance’ to mixing methods; this approach affirms that bringing together multiple theoretical paradigms strengthens findings, offering a more nuanced understanding of the object of enquiry [20, 21]. In this case, the methods were conducted independently and concurrently, and the results were integrated only at the stage of interpretation [22]; that is, neither method was used to inform the conduct of the other and so the results are discussed in relation to each other in the Discussion. This approach aimed to preserve the integrity of each discrete method.

####  Quantitative Component

 The quantitative study design and methods have been outlined in previous publications [23–25]. In short, data were collected as part of the PrEPARE Project, a repeated, national, cross-sectional survey of GBQ + people. The survey has been conducted online, biennially and around the same time of year since 2011. For the 2023 survey round, data were collected in June–July using Qualtrics online survey software (Provo, UT). We promoted the survey through paid advertisements on Facebook and Instagram and the dating/hook up app Grindr. Participants who provided consent in 2021 to be contacted about the 2023 survey were emailed an invitation to participate. Participants of a national mpox survey conducted in 2022 who provided consent to be contacted about sexual health and HIV prevention research were also emailed an invitation to the survey [26]. All potential participants were directed to a Qualtrics landing page, which provided information about the study. Participants provided consent before starting the survey. Eligible participants were aged 16 years or older, did not identify as female, did not identify as heterosexual, and lived in Australia; that is, male or non-binary and gay, bisexual or queer-identified people could participate. Ineligible participants were screened out automatically if they did not meet any of the inclusion criteria. Standard data cleaning procedures involved the removal of incomplete surveys (*n* = 511), and responses with excessive missing data (*n* = 4) and excessive flatlining on scales (*n* = 7). There was no evidence of fraudulent or bot responses, which were further minimised as participants were not compensated financially. The study was approved by the ethics committee of UNSW Sydney (HC230024) and the community organization ACON’s research review panel (202301).

####  Qualitative Component

Qualitative data were derived from a broader cognitive interviewing study that evaluated changes to an unrelated HIV and sexual health questionnaire. Cognitive interviewing involves presenting research participants with a survey questionnaire and discussing it during an interview to investigate whether questions are comprehended and fulfill their intended purposes [27, 28]. Fourteen participants were shown two prevention-effective adherence questions to provide feedback, including the original item and a newly developed item on partner risk factors. Participants were recruited in August–October 2023 with assistance from community-based partners who shared advertisements and flyers among their networks and on social media. All potential participants were directed to complete an expression of interest form to ensure a demographically diverse sample and to screen out potential fraudulent respondents [29]. Eligible participants were aged 18 years or older, spoke English, lived in Australia, and met one of the following criteria: they were gay, bi, or queer men (cisgender or transgender); a man who had had sex with men in the past 5 years; or a non-binary person who had had sex with gay, bi, or queer men in the past 5 years. The difference in age limits for participation in the quantitative vs. qualitative studies was due to online vs. in-person nature of each method, respectively. We decided minors would not be directly approached by researchers whereas online participation included participants aged 16–17 years because it allowed for private participation at a time and location of their choosing, with the ability to withdraw by closing their web browser. On interview completion, participants were offered AUD$50 gift card compensation in recognition of their time. Participants provided written or audio-recorded verbal consent prior to interviews. The study was approved by the ethics committee of UNSW Sydney (HC230384) and the community research review panels of ACON (202323) and Thorne Harbour Health (23010).

### Measures

####  Quantitative Component

The questionnaire measures have been described elsewhere [23, 24]. We collected data on demographics, recent sexual practices, HIV status and testing, STI testing and recent diagnoses, use of HIV treatment and PrEP, and attitudes and preferences toward taking PrEP. The variables and their categories are shown in the reporting.

For the primary outcome, prevention-effective adherence was measured using the single item developed by Fraser and colleagues [16]: ‘How many times in the last 6 months did you have sex without a condom that wasn’t covered by you using PrEP?’ An explanatory note was shown alongside the item: ‘“Not covered by PrEP” means you missed more than one dose or had stopped taking PrEP when you had sex’. Response options were ‘None’, ‘1–2’, ‘3–5’, ‘6–10’, and ‘More than 10’. Responses other than ‘None’ were coded as having had any PrEP-unprotected CLAIC. To assess ‘prevention by proxy’, participants who reported any PrEP-unprotected CLAIC were shown an additional matrix-style item, which was newly developed for this study, asking about potential risk reduction strategies used by participants and/or their partners: ‘When you had sex without a condom that wasn’t covered by PrEP, what did you know about your partner(s)?’ Strategies were: ‘They were on PrEP’; ‘They had an undetectable viral load’; and ‘They were HIV-negative’. Another response option captured whether participants had partners of an unknown HIV status (‘I didn’t know their HIV status’). For each strategy, participants responded with: ‘None’, ‘1–2’, ‘3–5’, ‘6–10’, or ‘More than 10’. The phrasing, ‘what did you *know* about your partner’, was deliberately used to account for both verbal and non-verbal disclosure about HIV status and prevention; that is, to ensure that direct discussion and indirect disclosure via a dating/hookup app were included. Adaptive survey logic was used so that the highest available category matched responses to the previous question (e.g., people who had 1–2 episodes of PrEP-unprotected CLAIC were only shown ‘None’ and ‘1–2’). The item did not capture any partners with a known detectable viral load.

Ease of getting PrEP and experience of side effects were measured with two items, ‘Getting PrEP is easy’ and ‘I get side effects from taking PrEP’, with response options ranging from Strongly disagree (1) to Strongly agree (5). Responses were dichotomized for use in the regression models (Agree/Strongly agree vs. the other options). Preferences for PrEP modality were measured using an established two-step approach [30, 31] that first asked which PrEP modalities participants would want to use: ‘New forms of PrEP are emerging or being developed. If all of these options were available (and equally effective in preventing HIV), which of these would you want to use? (select all that apply)’. Response options were: ‘Daily pill’, ‘Pills before and after sex (event-based or ‘on-demand’ dosing)’, ‘Monthly pill’, ‘Long-acting injection (e.g. every two months)’, ‘Long-acting, removable implant (under the skin)’, ‘Other (please specify)’, and ‘None of the above’. Participants could select multiple modalities during the first step, and then their selections in the first item were carried forward as the response options to the second question: ‘Which would be your most preferred way of taking PrEP?’, where they could choose one modality only. If participants only chose one option in the first step, that option was coded as their most preferred method, and they were not shown the second question.

####  Qualitative Component

 Audio-recorded interviews were undertaken by video conferencing or telephone. Participants were asked to view the prevention-effective adherence questions. Participants were provided a copy of the two questions on screen or sent a copy via email, and they were asked to read the question out loud and answer it for themselves or hypothetically. They were then asked to provide feedback on their comprehension of the question, with general probes such as ‘Why do you think these questions are asked?’, and ‘How did you come up with your answers?’ [27]. Demographic characteristics were collected following the interview, and participants chose or asked the interviewer to choose a pseudonym.

### Analyses

#### Quantitative Component

 Frequencies and proportions are reported for demographic and behavioral variables. Bivariable associations were analyzed using Pearson’s chi-squared tests and logistic regression. Multivariable logistic regression was used to identify independent associations of demographic and behavioral variables with the dichotomized outcome measure of prevention-effective adherence (only PrEP-protected CLAIC vs. some PrEP-unprotected CLAIC). Independent variables that were statistically significant at the bivariable level were block entered into the multivariable analysis. For a sensitivity analysis, the variables included in the primary model were assessed at both the bivariable and multivariable level with an alternative outcome measure (no or infrequent PrEP-unprotected CLAIC vs. at least 3 episodes of PrEP-unprotected CLAIC). Variables in the regression models had no missing observations. We report unadjusted and adjusted odds ratios (OR and aOR) with 95% confidence intervals (CI). Statistical significance was set at *p* < .05 (two-tailed). Analyses were conducted using Stata version 17.1 (StataCorp, College Station, TX).

####  Qualitative Component

 The analysis of the cognitive interviews focused on assessing problems with comprehension or item vagueness [27]. Interview recordings were transcribed, and excerpts relating to the prevention-effective adherence questions were extracted and then manually coded and organized in Microsoft Word to identify common problems with the questions, while also noting when participants reported that the questions were easily comprehensible. This analysis presents the range of responses and suggestions to improve the questions, with illustrative quotes.

## Results

### Quantitative Component

A total of 2,046 participants completed the survey in 2023. The median age was 35 years (IQR = 27–46). Most identified as gay (*n* = 1,672; 81.7%), were born in Australia (*n* = 1,430; 69.9%), were university educated (*n* = 1,309; 64.0%), and lived in the capital city of their state or territory (*n* = 1,579; 77.2%). Most were cisgender male (*n* = 1,944; 95.0%); smaller proportions were transgender male (*n* = 44; 2.2%) or non-binary (*n* = 58; 2.8%). Most participants lived in one of Australia’s three most populous states: New South Wales (*n* = 663; 32.4%), Victoria (*n* = 568; 27.8%), or Queensland (*n* = 380; 18.6%). Most participants reported full-time employment (*n* = 1,391; 68.0%). Most were HIV-negative (*n* = 1,699; 83%), with smaller proportions of untested participants (*n* = 208; 10.2%) and participants living with HIV (*n* = 139; 6.7%). Of the whole sample, 1,194 (58.4%) had ever used PrEP and 46.3% (*n* = 947) were current PrEP users. More than two thirds of the sample had been tested for HIV (*n* = 1,476; 72.1%) or other sexually transmissible infections (*n* = 1,444; 70.6%) within the 12 months preceding the survey. Most participants were recruited via advertising on Meta platforms, Facebook and Instagram (*n* = 1,116, 54.5%), or Grindr (*n* = 246, 12.0%). Less than one third of participants were recruited via direct email invitation (*n* = 638; 31.2%; i.e., confirmed repeat participants from a prior study wave). The remaining participants were recruited via Twitter, WeChat and the study website (*n* = 46; 2.2%).

#### Current PrEP User Characteristics

 The median age of the 947 current PrEP users was 37 years (IQR = 29.0–47.0). Like the broader sample, most identified as gay (*n* = 821; 86.7%), were Australian born (*n* = 688; 72.7%), university educated (*n* = 665; 70.2%), and lived in the capital city of their state or territory (*n* = 782; 82.6%). Most were cisgender male (*n* = 918; 96.9%); the remaining proportions were transgender male (*n* = 11; 1.2%) or non-binary (*n* = 18; 1.9% [15/18 were assigned male at birth]). Most current PrEP users were taking PrEP daily (*n* = 612; 64.6%), followed by event-based ‘2-1-1’ dosing (*n* = 297; 31.5%), periodic dosing (*n* = 33; 3.5%), or they were taking PrEP another way (*n* = 5; 0.5%). Nearly all PrEP users had been tested for HIV (*n* = 926; 97.8%) or other sexually transmissible infections (*n* = 884; 93.3%) within the previous 12 months. Most current PrEP users had only had sex with male partners in the six months preceding the survey (*n* = 830; 87.6%; inclusive of casual and regular partners). In relation to casual sex partners, 881 (93.0%) had casual male partners, 64 (6.8%) had casual non-binary partners, and 27 (2.9%) had casual female partners. Restricting PrEP users’ sexual partnership types and risk behavior to mutually exclusive categories, 10 (1.1%) had no sexual partners, 54 (5.7%) had regular partners only, 38 (4.0%) had casual partners but no penetrative sex, 53 (5.6%) had casual partners and consistently used condoms, 598 (63.2%) had CLAIC protected by their own PrEP use, and 194 (20.5%) had any PrEP-unprotected CLAIC. That is, of the 947 PrEP users, 20.5% had PrEP-unprotected CLAIC, and of the 792 PrEP-using participants who reported any CLAIC, 24.5% had PrEP-unprotected CLAIC.

Among the 194 PrEP users who reported PrEP-unprotected CLAIC in the last six months, 132 (68.0%) reported 1–2 episodes, 37 (19.1%) reported 3–5 episodes, 8 (4.2%) reported 6–10 episodes, and 17 (8.8%) reported > 10 episodes. Among the same group, 23 (11.9%) reported PrEP-unprotected CLAIC limited to PrEP users or people with undetectable viral loads, while 171 (88.1%) reported ≥ 1 PrEP-unprotected CLAIC episode with an assumed HIV-negative, i.e., unknown-status partner. Among the 194 participants who reported PrEP-unprotected CLAIC, the most preferred ways to take PrEP were monthly pills (*n* = 59; 30.4%), long-acting injections (*n* = 53; 27.3%), on-demand PrEP (*n* = 34; 17.5%), long-acting, removable implants (*n* = 32; 16.5%), and daily pills (*n* = 15; 7.7%); one participant wrote ‘vaccine’ as their preferred PrEP modality using a free-text box (0.5%). There were no statistically significant differences in preferred PrEP modalities between PrEP users who did or did not have PrEP-unprotected CLAIC (*X*^2^(6, 792) = 9.63, *p* = .14).

#### PrEP Prevention-Effective Adherence

 Current PrEP users who reported any CLAIC in the past six months were included in subsequent analyses (*n* = 792). Table 1 shows the sub-sample’s characteristics and factors associated with PrEP-protected CLAIC versus PrEP-unprotected CLAIC. Bivariable statistical differences were observed between the groups for: age, country/region of birth, employment status, PrEP dosing strategies, the duration participants were taking PrEP, self-rated ease in getting PrEP, PrEP side effects, and for reported drug-enhanced sex in the six months before the survey (See Table 1). Because nearly all of the sub-sample had been tested for HIV (*n* = 775; 97.9%) and for STIs (*n* = 742; 93.7%) in the 12 months preceding the survey, these variables were excluded from the analyses. None of the transgender men and non-binary participants in the sub-sample reported PrEP-unprotected CLAIC; gender experience (cisgender, transgender, non-binary) was therefore also excluded from the multivariable analysis. Lastly, the primary outcome measure was not associated with participants’ state/jurisdiction of residence (*X*^2^(3, 792) = 6.47, *p* = .091), a simplified measure of low income (<$40,000 vs. ≥$40,000 AUD; *X*^2^(1, 792) = 1.21, *p* = .271), nor with where participants sourced their PrEP; e.g., from an Australian pharmacy (*X*^2^(1, 792) = 1.39, *p* = .238), or online from overseas (*X*^2^(1, 792) = 1.45, *p* = .229). These variables have therefore been excluded from the reporting.

**Table 1:** Factors associated with PrEP-protected CLAIC vs. any PrEP-unprotected CLAIC among 792 participants

	Total	Only PrEP-protected CLAIC	Any PrEP-unprotected CLAIC	OR (95% CI)	*p* value	aOR (95% CI)	*p* value
	*n* = 792	*n* = 598	*n* = 194				
Age (years)							
≥ 30	601 (75.9)	477 (79.8)	124 (63.9)	Ref		Ref	
< 30	191 (24.1)	121 (20.2)	70 (36.1)	2.23 (1.56–3.17)	< 0.001	1.91 (1.27–2.88)	**0.002**
Sexuality							
Gay	687 (86.7)	514 (86.0)	173 (89.2)	Ref			
Bi+/Queer/Another term	105 (13.3)	84 (14.0)	21 (10.8)	0.74 (0.45–1.23)	0.252		
Residential location							
Inner metro	447 (56.4)	333 (55.7)	114 (58.8)	Ref			
Outer metro	215 (27.1)	162 (27.1)	53 (27.3)	0.96 (0.66–1.39)	0.813		
Regional and remote	114 (14.4)	89 (14.9)	25 (12.9)	0.82 (0.50–1.34)	0.431		
No response	16 (2.0)	14 (2.3)	2 (1.0)	0.42 (0.09–1.86)	0.252		
Country of birth							
Australia	583 (73.6)	456 (76.3)	127 (65.5)	Ref		Ref	
Asia	75 (9.5)	45 (7.5)	30 (15.5)	2.39 (1.45–3.95)	0.001	2.38 (1.28–4.42)	**0.006**
Europe	64 (8.1)	49 (8.2)	15 (7.7)	1.10 (0.60–2.02)	0.762	1.37 (0.70–2.67)	0.356
Other	70 (8.8)	48 (8.0)	22 (11.3)	1.65 (0.96–2.83)	0.071	1.80 (0.96–3.39)	0.069
Education level							
High school/Trade certificate	231 (29.2)	169 (28.3)	62 (32.0)	Ref			
University degree	561 (70.8)	429 (71.7)	132 (68.0)	0.84 (0.59–1.19)	0.325		
Employment status							
Employed full-time	622 (78.5)	479 (80.1)	143 (73.7)	Ref		Ref	
Employed part-time	98 (12.4)	61 (10.2)	37 (19.1)	2.03 (1.30–3.18)	0.002	1.67 (1.01–2.75)	**0.044**
Student/unemployed/other	72 (9.1)	58 (9.7)	14 (7.2)	0.81 (0.44–1.49)	0.497	0.68 (0.33–1.40)	0.293
Medicare ineligible	48 (6.1)	30 (5.0)	18 (9.3)	1.94 (1.05–3.56)	0.033	0.72 (0.32–1.63)	0.429
PrEP dosing strategy							
Current daily user	519 (65.5)	434 (72.6)	85 (43.8)	Ref		Ref	
Current on-demand user	246 (31.1)	149 (24.9)	97 (50.0)	3.32 (2.35–4.70)	< 0.001	2.73 (1.88–3.96)	**< 0.001**
Current periodic user	27 (3.4)	15 (2.5)	12 (6.2)	4.08 (1.85–9.04)	0.001	3.50 (1.39–8.83)	**0.008**
Time since first taking PrEP							
One year or more	625 (78.9)	489 (81.8)	136 (70.1)	Ref		Ref	
Less than one year	167 (21.1)	109 (18.2)	58 (29.9)	1.91 (1.32–2.77)	0.001	1.69 (1.12–2.56)	**0.013**
Number of male sex partners (last 6 months)							
1–5	238 (30.1)	180 (30.1)	58 (29.9)	Ref			
6–10	213 (26.9)	167 (27.9)	46 (23.7)	0.85 (0.55–1.33)	0.485		
>10	341 (43.1)	251 (42.0)	90 (46.4)	1.11 (0.76–1.63)	0.583		
Getting PrEP is easy	585 (73.9)	465 (77.8)	120 (61.9)	0.46 (0.33–0.66)	< 0.001	0.62 (0.42–0.92)	**0.017**
I get side effects from taking PrEP	108 (13.6)	58 (9.7)	50 (25.8)	3.23 (2.12–4.92)	< 0.001	2.47 (1.57–3.90)	**< 0.001**
Drug-enhanced sex (last 6 months)	172 (21.7)	120 (20.1)	52 (26.8)	1.46 (1.00-2.12)	0.049	1.84 (1.21–2.79)	**0.004**

Data are n(%), and unadjusted odds ratios (OR) and adjusted odds ratios (aOR) with 95% confidence intervals (CI) derived from logistic regression. Bolded values show statistical significance at *p* < .05 for ease of reference. Except where stated, proportions represent affirmative (‘Yes’) responses. Bivariable analyses for state/territory and education were not significant, and were therefore excluded from the multivariable analysis (model *X*^2^ *p* values exceeded 0.05)

Table 1 shows the results of the primary multivariable analysis (*N* = 792). Participants who reported PrEP-unprotected CLAIC were more likely to: be < 30 years old compared to older (*aOR* = 1.91, 95%CI: 1.27, 2.88, *p* = .002), be born in Asia compared to Australia (*aOR* = 2.38, 95%CI: 1.28, 4.42, *p* = .006), be part-time employed compared to full-time employed (*aOR* = 1.67, 95%CI: 1.01, 2.75, *p* = .044), use on-demand (*aOR* = 2.73, 95%CI: 1.88, 3.96, *p* < .001) or periodic PrEP (*aOR* = 3.50, 95%CI: 1.39, 8.83, *p* = .008) compared to daily pills, be taking PrEP for less than one year versus longer (*aOR* = 1.69, 95%CI: 1.12, 2.56, *p* = .013), have experienced side effects from PrEP (*aOR* = 2.47, 95%CI: 1.57, 3.90, *p* < .001), and report any sexualized drug use in the past 6 months (*aOR* = 1.84, 95%CI: 1.21, 2.79, *p* = .004). They were less likely to find it easy to get PrEP (*aOR* = 0.62, 95%CI: 0.42, 0.92, *p* = .017).

Table 2 shows the results of the supplementary sensitivity analysis (*N* = 792). At the bivariable level, more frequent PrEP-unprotected CLAIC (at least 3 episodes in the last 6 months) was only associated with participants’ PrEP dosing strategy and their experience of side effects from PrEP. Multivariable analysis found that only participants’ PrEP dosing strategy was independently related to the alternative outcome measure; that is, when controlling for participants’ experience of side effects from PrEP, those who used PrEP on demand (*aOR* = 2.47, 95%CI: 1.42, 4.30, *p* = .001) or periodic PrEP (*aOR* = 4.84, 95%CI: 1.32, 11.02, *p* = .012) had greater odds of reporting more frequent PrEP-unprotected CLAIC compared to daily PrEP users.

**Table 2:** Sensitivity analysis of factors associated with PrEP-protected CLAIC and irregular PrEP-unprotected CLAIC vs. frequent PrEP-unprotected CLAIC among 792 participants

	Total	No / irregular PrEP-unprotected CLAIC	Frequent PrEP-unprotected CLAIC	OR (95% CI)	*p* value	aOR (95% CI)	*p* value
	*n* = 792	*n* = 730	*n* = 62				
Age (years)							
≥ 30	601 (75.9)	560 (76.7)	41 (66.1)	Ref			
< 30	191 (24.1)	170 (23.3)	21 (33.9)	1.69 (0.97–2.93)	0.064		
Sexuality							
Gay	687 (86.7)	634 (86.8)	53 (85.5)	Ref			
Bi+/Queer/Another term	105 (13.3)	96 (13.2)	9 (14.5)	1.12 (0.54–2.35)	0.761		
Residential location							
Inner metro	447 (56.4)	412 (56.4)	35 (56.5)	Ref			
Outer metro	215 (27.1)	197 (27.0)	18 (29.0)	1.08 (0.59–1.95)	0.81		
Regional and remote	114 (14.4)	106 (14.5)	8 (12.9)	0.89 (0.40–1.97)	0.771		
No response	16 (2.0)	15 (2.1)	1 (1.6)	0.78 (0.10–6.12)	0.817		
Country of birth							
Australia	583 (73.6)	541 (74.1)	42 (67.7)	Ref			
Asia	75 (9.5)	65 (8.9)	10 (16.1)	1.98 (0.95–4.14)	0.069		
Europe	64 (8.1)	60 (8.2)	4 (6.5)	0.86 (0.30–2.48)	0.778		
Other	70 (8.8)	64 (8.8)	6 (9.7)	1.21 (0.49–2.95)	0.679		
Education level							
High school/Trade certificate	231 (29.2)	212 (29.0)	19 (30.6)	Ref			
University degree	561 (70.8)	518 (71.0)	43 (69.4)	0.93 (0.53–1.63)	0.79		
Employment status							
Employed full-time	622 (78.5)	575 (78.8)	47 (75.8)	Ref			
Employed part-time	98 (12.4)	86 (11.8)	12 (19.4)	1.71 (0.87–3.35)	0.119		
Student/unemployed/other	72 (9.1)	69 (9.5)	3 (4.8)	0.53 (0.16–1.75)	0.3		
Medicare ineligible	48 (6.1)	43 (5.9)	5 (8.1)	1.40 (0.53–3.68)	0.493		
PrEP dosing strategy							
Current daily user	519 (65.5)	493 (67.5)	26 (41.9)	Ref		Ref	
Current on-demand user	246 (31.1)	215 (29.5)	31 (50.0)	2.73 (1.58–4.72)	< 0.001	2.47 (1.42–4.30)	**0.001**
Current periodic user	27 (3.4)	22 (3.0)	5 (8.1)	4.31 (1.51–12.29)	0.006	3.84 (1.34–11.02)	**0.012**
Time since first taking PrEP							
One year or more	625 (78.9)	580 (79.5)	45 (72.6)	Ref			
Less than one year	167 (21.1)	150 (20.5)	17 (27.4)	1.46 (0.81–2.63)	0.205		
Number of male sex partners (last 6 months)							
1–5	238 (30.1)	219 (30.0)	19 (30.6)	Ref			
6–10	213 (26.9)	197 (27.0)	16 (25.8)	0.94 (0.47–1.87)	0.852		
>10	341 (43.1)	314 (43.0)	27 (43.5)	0.99 (0.54–1.83)	0.977		
Getting PrEP is easy	585 (73.9)	544 (74.5)	41 (66.1)	0.67 (0.38–1.16)	0.151		
I get side effects from taking PrEP	108 (13.6)	92 (12.6)	16 (25.8)	2.41 (1.31–4.44)	0.005	1.85 (1.00-3.46)	0.052
Drug-enhanced sex (last 6 months)	172 (21.7)	156 (21.4)	16 (25.8)	1.28 (0.71–2.32)	0.417		

Data are n(%), and unadjusted odds ratios (OR) and adjusted odds ratios (aOR) with 95% confidence intervals (CI) derived from logistic regression. Frequent PrEP-unprotected CLAIC included at least 3 episodes of PrEP-unprotected CLAIC in the last 6 months. Bolded values show statistical significance at *p* < .05 for ease of reference. Except where stated, proportions represent affirmative (‘Yes’) responses. Bivariable analyses for state/territory and education were not significant, and were therefore excluded from the multivariable analysis (model *X*^2^ *p* values exceeded 0.05)

### Qualitative Component

A total of 30 participants were interviewed, and 14 were presented the prevention-effective adherence questions, e.g., ‘How many times in the last 6 months did you have sex without a condom that wasn’t covered by you using PrEP?’ and the prevention by proxy question (see above on quantitative measures for full wording of questions). Table 3 shows participant characteristics for the 14 participants. In summary, most identified as cisgender men (71.4%), half were gay (50%) and smaller proportions were bi + or queer; and most were aged in their 30s (64.3%). Half of the participants reported Australia as their country of birth (50%), and four reported a non-Anglo/white ethnic background. More than half of the participants were current PrEP users (57.1%), including two on-demand users. Most interviews were conducted via video conferencing, with one by telephone.

**Table 3:** Interview participant characteristics

Characteristics	*N* = 14
Gender	
Cisgender men	10
Non-binary	2
Transgender men	1
Genderqueer	1
Sexual Orientation	
Gay	7
Bi, bisexual and/or pansexual	4
Queer	2
Queer/bisexual	1
Mean age	33
Age (groups)	
20s	3
30s	9
40s	1
50s	1
State	
New South Wales	9
Victoria	3
Australian Capital Territory	1
Queensland	1
HIV status and ARV use	
Living with HIV, undetectable viral load	1
HIV-negative, not using PrEP	5
HIV-negative, daily PrEP	6
HIV-negative, on-demand PrEP	2
Country of Birth	
Australia*	7
Aotearoa New Zealand	2
South-East Asia	2
Southern and Central Asia	2
United Kingdom	1

Data are frequencies except where indicated. *Among those born in Australia, 4 reported non-Anglo or white ethnic backgrounds, including from Europe, the Middle East, South America, Central Asia, and South-East Asia. No participants identified as Aboriginal or Torres Strait Islander

All participants indicated that they could answer the questions and interpret what was being asked of them. Five participants had no specific feedback, and the rest provided minor comments about how to improve clarity and reduce confusion. Illustrative quotes can be found in Table 4. The primary feedback provided by participants related to the vague or ambiguous meaning of the word ‘covered’. Some argued that the phrase was unfamiliar, or that the wording could be simplified to be more direct. Other feedback included that using the word ‘covered’ as well as ‘condoms’ in the same sentence created confusion because the question was asking about PrEP coverage, but the word ‘covered’ might imply condom use. Participants who were using PrEP indicated that they could answer the questions but found the phrase ‘covered’ to be unfamiliar.

**Table 4:** Types of responses with illustrative quotes

Type	Illustrative Quotes
No issues	“They’re really good questions, easy to answer.” *(Logan*,* non-binary/trans femme queer person*,* Daily PrEP user)* “[First question] I’ll put none, yeah. And the second one, I would put like none as well, I think.” *(Brin*,* gay cis man*,* Daily PrEP user)* “‘Not covered by PrEP’ makes sense. […] I’ve definitely missed a few doses here and there, but never consecutively two in a row and I haven’t had sex on those days. I tend to find it’s like the middle of the week, I’m drowning in work, and I just don’t remember.” *(Thomas*,* gay cis man*,* Daily PrEP user)* “It’s good that you give a definition of “not covered by PrEP.” I have sometimes missed my dosages, probably six times in four or five years, but I never had sex, though, in those periods, but I was told by my doctor that that was fine and there was no issue with that at all. *(Hobart*,* bisexual/pansexual cis man*,* Daily PrEP user)*
Item vagueness – ‘covered’	“When I was taking it daily, even if I missed, let’s say a dose or two doses, I was technically still covered by PrEP. I don’t know if ‘covered’ is the right word.” *(Michael*,* gay cis man*,* not using PrEP)* “The word ‘covered’ is interesting. That’s not a term that I’ve ever heard used before in the community. I’m just trying to think if there’s a different way to say that’s more clear… […] it’s the word ‘cover’ also with the word ‘condom’ when in the context of the same sentence.” *(Will*,* genderqueer bisexual/queer person*,* Daily PrEP user)* “Something about the words like ‘not covered by PrEP.’ I don’t know why. It’s just there’s something about the wording, it seems odd.” *(Nick*,* bisexual man of trans experience*,* not using PrEP)* “My initial response when I first read it was the language around ‘covered,’ as in ‘covered by a condom, covered by PrEP’, it just reads strangely. […] I almost think it should just be reworded and have less words somehow […] ‘was there an occasion where you had sex and you didn’t use a condom and you weren’t using the PrEP?’” *(Peter*,* gay cis man*,* on-demand PrEP user)* “Maybe just simplifying it to be like ‘How many times in the last six months did you have sex without a condom and weren’t taking PrEP or and didn’t take PrEP?’ maybe.” *(Vinh*,* non-binary queer person*,* on-demand PrEP user)*

## Discussion

Using national, cross-sectional survey data, we trialed a brief self-report measure of prevention-effective adherence to HIV PrEP among gay, bisexual and queer men and non-binary people in Australia. The interview findings suggested that while using the word ‘covered’ in the context of PrEP use was unfamiliar, participants found the items intelligible and were able to complete them. The survey findings indicated that prevention-effective adherence was 79.5% among all PrEP users in the last six months; that is, one fifth of PrEP users reported one or more episodes of PrEP-unprotected CLAIC. Most PrEP users who had any CLAIC engaged in few PrEP-unprotected episodes: 24.5% [194/792] had at least one episode of PrEP-unprotected CLAIC in the past six months, but 7.8% [62/792] had three or more episodes of PrEP-unprotected CLAIC. Among those who reported any PrEP-unprotected CLAIC, nearly 90% had at least one episode of PrEP-unprotected CLAIC with a partner of unknown HIV status (or assumed HIV-negative status), suggesting that the deliberate use of prevention by proxy methods to reduce risk during these encounters was uncommon.

Very few quantitative methods of measuring prevention-effective adherence have been published in the literature to date. A key aim of this study was to assess individuals’ feedback on the prevention-effective adherence measure. The questions appeared to be understood and answerable by participants, although some interview participants indicated that the phrasing ‘covered by PrEP’ was unfamiliar and potentially confusing while also mentioning condoms in the same sentence. In response to the cognitive interview findings, and in consultation with our community organization reference group, we altered the measure’s phrasing in a subsequent study wave, from ‘covered by PrEP’ to ‘protected by PrEP’, and we recommend such wording be considered by other researchers who opt to use this measure in the future. Further research should consider how well the items can be translated into other languages, and their use with populations with low English-language fluency.

Given the differing methods of quantitatively measuring prevention-effective adherence, comparisons between studies are challenging. We define such a method as one that measures the timing of PrEP pill taking and sexual behavior in a way that can quantify acts (the number or proportion) of PrEP-unprotected CLAIC. Our estimate of 20.5% PrEP users reporting one or more acts of PrEP-unprotected CLAIC was lower than the first Australian estimate using the same measure (46.5%) [16]. This large difference may be attributed to our survey having a larger sample size of PrEP users drawn from all over Australia, differences in recruitment method (e.g. the previous study primarily relied on clinic-based recruitment), or slight differences in the wording of the question.

Another Australian study used a very different method to quantify prevention-effective adherence in the context of a large oral PrEP demonstration trial, and collected data on sexual behavior and PrEP pill-taking in the week prior to each quarterly survey [7]. This approach found that only 1% of examined weeks involved suboptimal PrEP coverage during potential risk episodes. While this varies significantly from our current estimate, the vastly different method means that it is somewhat inappropriate to compare them. In addition, the ‘last full week’ method was not able to account for pill taking and sexual behaviors over a longer recall period but rather provided a more recent and shorter snapshot of time. A cohort study in Taiwan measured participants’ PrEP use within five days around the most recent episode of anal intercourse in the past month and found that PrEP was not taken correctly at 15.8% of visits; however, it is unclear if this was only for acts of CLAIC or any anal intercourse [32]. Longitudinal studies of prevention-effective adherence may be prone to bias due to loss-to-follow-up and may underestimate PrEP-unprotected CLAIC. In any case, it is clear that more data are needed on these various methods to measure prevention-effective adherence, ideally comparing multiple methods head-to-head in the same study.

Our analysis found several demographic and behavioral characteristics were associated with PrEP-unprotected CLAIC, such as younger age, which has been well-established in the literature as being associated with lower PrEP use, prevention-effective adherence, and PrEP persistence [7, 13, 16, 17]. We found that part-time employment was associated with PrEP-unprotected CLAIC, consistent with the previous Australian study using this method [16]. We also found that Asian-born participants (vs. Australian-born) were more likely to report PrEP-unprotected CLAIC, which may reflect disparities in the HIV epidemic that have emerged over the past decade in Australia, whereby HIV diagnoses among Australian-born MSM have decreased substantially but diagnoses among Asian-born MSM have not [33]. Indeed, in 2023, 36% of national HIV notifications in MSM were among those born in Asia [33]. PrEP-unprotected CLAIC was higher among those reporting any sexualized drug use, which is consistent with prior research from Australia in relation to the association between recreational drug use and poorer PrEP adherence [12, 13].

Several PrEP-related factors were also highlighted in our multivariable analyses, whereby non-daily PrEP users, PrEP users with less than one year of experience taking PrEP, and those who had experienced PrEP-related side effects were more likely to report PrEP-unprotected CLAIC, while those who found it easy to get PrEP were less likely. Prior research has found that some PrEP users have complex patterns of PrEP initiation, suspension, and recommencement, and they may switch between daily and non-daily PrEP dosing strategies [34]. The same study highlighted difficulties that some men had navigating sex after discontinuing or suspending PrEP, particularly in terms of having unanticipated PrEP-unprotected CLAIC in which they did not feel comfortable negotiating condom use, either due to the “heat of the moment” or pressure from sex partners not to use condoms [34]. While tempting to suggest that HIV prevention campaigns reinforce condom use in the absence of PrEP, the observed changes in relation to social norms and condom (non-)use may mean it is more expedient to address structural barriers to PrEP adherence; for example, health authorities could consider alternative PrEP provisioning options so that individuals do not need to visit multiple sites to get PrEP medication (a clinic, pathology and pharmacy), or streamline ways to access repeat scripts [35].

The sensitivity analysis we conducted (examining associations with *frequent* PrEP-unprotected CLAIC) showed that participants’ use of non-daily PrEP dosing strategies and the experience of PrEP-related side effects were associated with more frequent PrEP-unprotected CLAIC, however, only non-daily dosing strategies were independently associated with more frequent PrEP-unprotected CLAIC. This result suggests that the experience of side effects has an indirect effect on PrEP adherence, as side effects appear to influence the choice of dosing strategy, which in turn may lead to an increased likelihood of suboptimal adherence during sex [36]. Here, it is worth noting that while most PrEP users (68.8%) who did not report PrEP-related side effects took PrEP daily, most participants (60.4%) who had experienced side effects were on-demand or periodic PrEP users. Previous studies have also shown that side effects influence PrEP discontinuation among MSM [34, 37]. Future research should explore the relationship between PrEP side effects, dosing strategy and prevention-effective adherence. Additionally, as long-acting PrEP formulations become available, they may offer a promising solution to improve prevention-effective adherence by reducing the burden of daily pill-taking and potentially mitigating the impact of side effects. However, the introduction of these new formulations should be accompanied by efforts to understand and address user preferences and potential access barriers. It is worthwhile noting that the majority of those who reported PrEP-unprotected CLAIC preferred long-acting forms of PrEP, including monthly pills (30.4%) or long-acting injections (27.3%).

## Limitations

This study has several limitations. First, the data were self-reported and may be subject to social desirability and recall biases, although it is likely that anonymous participation in the survey may have mitigated the first issue. We designed the self-report measure of prevention-effective adherence to PrEP to overcome some of the challenges of more direct measurement tools, such as the use of biological samples to verify PrEP use and adherence, which are costly and complex to implement. There are limited Australian data on concordance between self-reported PrEP adherence and biological measures; however, one analysis from an Australian clinical trial (*n* = 89) found relatively high agreement for self-report (76%) and very high agreement for clinician-facilitated recall (96%) when compared to drug levels [38]. While our measure is inherently limited by its reliance on self-report, we believe the benefits – particularly scalability in resource-constrained settings – far outweigh these limitations. Second, the results are limited by the study’s non-random and convenience-based sample. We are unable to determine the representativeness of the sample because there are limited national data on gay, bisexual and queer men and non-binary people. Third, this study did not assess prevention-effective adherence among individuals who had previously used but discontinued PrEP. Future studies should consider including former PrEP users to better understand adherence lapses and transitions in prevention strategies. Finally, while cognitive interviewing provided valuable insights into participants’ understanding of the survey item, this process may have led to more carefully considered responses than typical survey responses [27].

## Conclusion

We have assessed a brief measure of prevention-effective adherence to HIV PrEP that warrants consideration for repeated use in HIV behavioral surveillance studies that measure PrEP uptake. While most PrEP users reported effective adherence to HIV PrEP, we found that one in five PrEP users reported any PrEP-unprotected CLAIC. A minority reported more frequent PrEP-unprotected CLAIC, which was associated with non-daily dosing. Targeted interventions to people who report more frequent PrEP-unprotected episodes must address side effects and other adherence barriers, and consider new PrEP modalities such as long-acting injections and streamlined provisioning for oral PrEP that address structural barriers to PrEP use.
